# Correlations Between Coronary Artery Calcium Scores and Vitamin A, the Triglyceride/High-Density Lipoprotein Ratio, and Glycated Hemoglobin in At-Risk Individuals in Saudi Arabia: A Comprehensive Cross-Sectional Study

**DOI:** 10.3390/jcm14113645

**Published:** 2025-05-22

**Authors:** Thamir Al-khlaiwi, Ayman Alsaleh, Fatimah Alghamdi, Farah Abukhalaf, Maryam Alghannam, Shahad Alzaid, Rahaf Alslimah, Reena Alsadouni, Hessah Alshammari

**Affiliations:** 1 Department of Physiology, College of Medicine, King Saud University, Riyadh 11461, Saudi Arabia; fatimalghamdi03@gmail.com (F.A.); farahabukhalaf443@gmail.com (F.A.); maryamalghannam443@gmail.com (M.A.); shahadalzaid8@gmail.com (S.A.); rahaffe3@gmail.com (R.A.); reenamed75@gmail.com (R.A.); 2 Department of Cardiac Sciences, King Fahad Cardiac Center, College of Medicine, King Saud University Medical City, King Saud University, Riyadh 11362, Saudi Arabia; aymalsaleh@ksu.edu.sa; 3 Department of Cardiac Sciences, College of Medicine, King Saud University, Riyadh 11461, Saudi Arabia

**Keywords:** coronary artery calcium score, vitamin A, glycated hemoglobin, triglyceride/high-density lipoprotein ratio, Saudi Arabia

## Abstract

**Background/Objectives**: Given the conflicting results and limited published data on the correlation of vitamin A, E, and D, parathyroid hormone (PTH), and thyroid-stimulating hormone (TSH) levels, the triglyceride to high-density lipoprotein (TG/HDL) ratio, and glucose levels with the coronary artery calcium score (CAC score) in individuals at risk of coronary artery disease (CAD), this relationship requires extensive investigation. Therefore, our study aimed to investigate the correlations between the aforementioned metrics and the CAC score in individuals at risk of CAD in Saudi Arabia. **Methods**: This analytical cross-sectional study was conducted at the Department of Physiology, College of Medicine at King Saud University Medical City (KSUMC), King Saud University, Riyadh, Saudi Arabia, between November 2024 and April 2025, targeting patients at risk of CAD. After recruiting patients from cardiology and primary care clinics, data regarding blood vitamin A, E, and D and PTH and TSH levels and CAC scores were collected from each patient’s electronic medical records. A score of 10 points was used as a cutoff between low and high CAC scores. **Results**: Our sample size was 172 patients. The majority of the patients were male (62.2%), and 37.8% were female. The mean age of the sample was 59.98 ± 9.26 years, with an age range spanning 40 years. Serum vitamin A levels had a significant negative correlation with CAC scores, (odds ratio (OR) = 0.147, *p*-value = 0.002), whereas vitamin D and E, PTH, and TSH levels did not correlate with this score. The TG/HDL ratio was positively and significantly correlated with CAC scores (OR = 1.654, *p*-value = 0.030). The analysis model showed that a patient’s mean serum glycated hemoglobin (HbA1c) level positively and significantly influenced their odds of having a high CAC score (OR = 1.364, *p*-value = 0.018). Patient ethnicity was not significantly associated with the CAC score (CAC ≥ 10 points) (*p* = 0.749). Similarly, BMI did not correlate with the CAC score (*p* = 0.722). However, male patients were 3.42 times more likely than females to have a high CAC score (CAC ≥ 10 points), a statistically significant difference (*p* = 0.005). No significant differences were observed between males and females in terms of their mean vitamin A (1.74 ± 0.58 vs. 1.80 ± 0.52, *p* = 0.633), vitamin E (41.41 ± 15.99 vs. 37.61 ± 11.78, *p* = 0.189), or vitamin D levels (80.35 ± 31.07 vs. 77.16 ± 26.15, *p* = 0.479). Additionally, the patient’s age was significantly positively associated with the likelihood of having a high CAC score, with OR = 1.102 times (*p* < 0.001). **Conclusions**: The findings of our study indicate the strong impact of vitamin A, the TG/HDL ratio, and HbA1c on CAC scores, among other factors affecting CAC scores, and they need more concern and attention. Understanding the cellular mechanism of vitamin A correlation with calcification is of great clinical value. The TG/HDL ratio is emerging as a novel index for CVD when compared to other lipid profile parameters. Intensive large-scale studies are needed to explore the interpretations as well as cutoff values of this valuable index. Males are more prone to CVD due to their high correlation with CAC scores. Therefore, vitamin A administration and strict HbA1c and TG/HDL ratio monitoring could help as prophylactic measures to prevent cardiovascular disease in these patients. These findings could influence specific preventive measures or screening strategies for cardiovascular disease in high-risk populations. A lifestyle medicine approach that involves caregivers as well as patients should be implemented to minimize the incidence and complications of detrimental diseases.

## 1. Introduction

Cardiovascular diseases (CVDs) are the leading cause of death worldwide, claiming approximately 17.9 million lives annually [1]. Among them, coronary artery disease (CAD) is one of the most prevalent and is the primary contributor to mortality and Disability-Adjusted Life Year (DALY) loss globally, particularly in low- and middle-income countries. CAD accounts for an estimated 7 million deaths and the loss of 129 million DALYs each year [2]. In 2015 alone, CAD was responsible for 8.9 million deaths and the loss of 164 million DALYs [3]. In Saudi Arabia, CAD is the leading cause of death, representing 24.3% of total fatalities [4]. Alarmingly, a study found that 5.5% of individuals aged 30 to 70 years in Saudi Arabia are affected by CAD [5]. It is well established that coronary atherosclerotic plaques are the main driver of CAD [6]. As the disease progresses, these plaques are closely associated with calcium accumulation in the arteries. Consequently, some studies suggest that detecting calcified deposits in the coronary artery walls may serve as an early indicator of CAD development [6]. The coronary artery calcium score (CAC score) uses cardiac CT to measure subclinical atherosclerosis through non-invasive tests that provide strong associations between cardiovascular risk and upcoming cardiac events [7,8]. There is no consensus regarding a cutoff value for the CAC score, with several studies suggesting different cutoffs. Kaczmarska et al. found a cutoff of 10 was correlated with high sensitivity [9] and revealed a progressive increase in CAD incidence as CAC scores increased. Notably, there was an approximately five-fold difference in CAD rates between individuals with CAC scores of 10 and those with scores ranging from 10 to 100 [9]. Cardiac gated multidetector computed tomographic scanners (MDCTs) allow for the quantification of CAC [10]. Non-gated studies can also assess CAC using either semiquantitative (ordinal scoring) or quantitative methods (Agatston scoring) and demonstrate a strong correlation with gated CT studies and cardiovascular disease outcomes [11,12]. Enhancing CAC assessment by combining CAC scores with blood biomarkers, genetic markers, or imaging features can improve the accuracy of cardiovascular risk evaluation, offering more precise risk stratification [13,14].

CAC scores have been reported to be strongly associated with factors such as age, hypertension, diabetes, and lifestyle behaviors [15]. While the associations between the mentioned factors and CAC scores are well established, the impact of vitamins A, E, and D, parathyroid hormone (PTH), and thyroid-stimulating hormone (TSH) on CAC scores remains uncertain. Vitamins are considered to play a role in the maintenance of cardiovascular health and alleviating CVD. This could be due to their antioxidant or other physiological effects that prevent atherosclerotic accumulation. Several studies have shown that deficiency in certain vitamins, such as A, E, and D, can contribute to cardiovascular problems [15]. Therefore, supplementing these vitamins is recommended to minimize the risk of CVDs, such as hypertension, atherosclerosis, myocardial ischemia, arrhythmia, and heart failure [15]. Given the conflicting results, as well as the limited published research on the correlation of vitamin A, E, and D, PTH, and TSH levels with CAC scores in individuals at risk of CAD, this relationship requires extensive investigation [16,17,18,19,20,21,22,23,24,25]. In addition, there are very limited studies regarding vitamins and their impact on CAC scores in Saudi Arabia, which might present special ethnic variations. Our research question was the following: Are vitamins A, E, and D deficiencies as well as PTH and TSH level disturbances associated with elevated calcium scores in subjects at risk of coronary artery disease in Saudi Arabia? Therefore, the primary objective of our study was to investigate the correlations of vitamins A, E, and D, PTH, TSH, the triglyceride to high-density lipoprotein (TG/HDL) ratio, and glucose levels with CAC scores in individuals at risk of CAD in Saudi Arabia, while the secondary objective was to provide comprehensive and clear insights into the role that these factors may play in cardiovascular risk and, hence, their inclusion in a lifestyle medicine approach. It is crucial that the findings of this study on a lifestyle medicine approach are applied to the control and prevention of risk factors, incidence, and complications of noncommunicable diseases that can be controlled through a multidisciplinary approach.

## 2. Methods

This analytical comprehensive cross-sectional study was conducted in the Department of Physiology, College of Medicine at King Saud University Medical City (KSUMC), King Saud University, Riyadh, Saudi Arabia, between November 2024 and March 2025, targeting patients at risk of coronary artery disease residing in Saudi Arabia. The independent variables included sociodemographic data, such as age, gender, and nationality, as well as vitamins (A, E, D), parathyroid hormone (PTH), thyroid-stimulating hormone (TSH), BMI, the lipid profile (triglyceride, HDL, LDL), the triglyceride/HDL ratio, liver function tests (ALT, AST, ALP), fibrinogen, creatinine, C-reactive protein, calcium, phosphate, glucose, and HbA1c. The outcome variable was the association of vitamin (A, E, D) levels as well as other variables with the CAC score. The inclusion criteria were patients at risk of CAD (who have a risk factor of CAD, such as hypertension, obesity, diabetes, and family history) in Saudi Arabia and patients aged between 18 and 80 years, whereas the exclusion criteria were patients who had confirmed CAD via CT angiography, patients residing outside Saudi Arabia, patients below the age of 18 years, and patients with congenital heart diseases.

**Figure 1:**
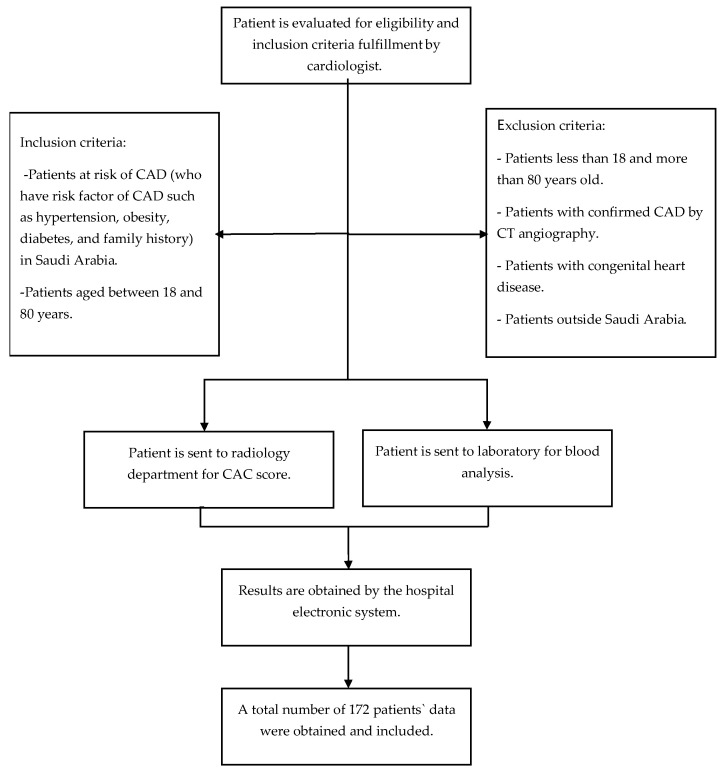
Flow diagram for the patient selection method.

After the patients were evaluated by a primary care physician and cardiologist to ascertain whether they fulfilled our inclusion criteria, they were sent to the Department of Radiology to obtain a CAC score using cardiac gated multidetector computed tomographic scanners (MDCTs) [10,11,12] with electrocardiographic (ECG) gating. Images were acquired with a slice thickness of 0.625 mm and reconstructed using an isotropic voxel resolution of 0.625 mm × 0.625 mm to ensure high spatial accuracy. Also, patients were sent to the hospital laboratory to undergo comprehensive blood tests for analysis of serum levels of vitamins A, E (using a Waters Alliance e2695 separation module, a 2489 UV/visible detector, and a 2475 fluorescence detector to perform high-performance liquid chromatography (HPLC), Waters, Milford, MA, USA), and D (using a Cobas e 801 analyzer by Roche Diagnostics to perform electrochemiluminescence (ECLIA), Roche, Vienna, Austria) and assess PTH, TSH, total cholesterol (TC), serum HDL, serum low-density lipoprotein (LDL), serum triglyceride (TG), the TG/HDL ratio, glucose, calcium, phosphate, creatinine, alkaline phosphatase (ALP), serum alanine aminotransferase (ALT), serum aspartate aminotransferase (AST), fibrinogen, serum glycated hemoglobin (HbA1c), and C-reactive protein (HsCRP) in the blood. The laboratory personnel were blinded to the clinical data. Regarding the demographic characteristics (age, gender, height, weight, body mass index (BMI), and nationality), blood vitamin A, E, and D, PTH, and TSH levels, biochemical biomarkers, and CAC scores were collected from the patients’ electronic medical records (eSIHI) (Figure 1). A CAC score of 10 points was used as a cutoff between low and high scores [9]. The sample size was calculated using the single proportion equation: n = Z^2^ × P(1 − P)/d^2^, where Z = 1.96 (95% confidence level), d = 0.05, and P = the proportion of subjects at risk, which was assumed to be 10% due to the lack of studies on subjects who are at risk of CAD [5] [(1.96^2^) × 0.1(1 − 0.1)/(0.05)^2^ = 138]. The calculated sample size was 138 patients. However, we recruited 172 patients. This study was approved by the Institutional Review Board (IRB), College of Medicine, King Saud University (No. E-24-9215), and conducted in accordance with the Declaration of Helsinki. The study maintained the privacy, confidentiality, and anonymity of the participants.

## 3. Statistical Analysis

The mean and standard deviation (SD) were used to describe the measured continuous variables, and the median and inter-quartile range were used to describe continuous variables that had statistical skewness. Frequencies and percentages were used to describe the categorical variables. The Kolmogorov–Smirnov statistical normality test and histograms were used to assess the statistical normality assumptions for the metric variables. The chi-squared test for independence was used to assess the correlations between categorically measured variables, and Pearson’s correlation test was used to assess the correlations between metric variables. The independent-samples *t*-test was used to assess the statistical significance of mean scores across the levels of binary categorical variables. Multivariable Binary Logistic Regression Analysis (MBLR) was applied to assess the predictors of a patient’s odds of having a high coronary calcium score (≥10 points) when regressed against their sociodemographic factors and other measured laboratory test findings and related factors. The relationships between the predictor variables and analyzed outcomes in MBLR were presented as multivariable-adjusted odds ratios (ORs) with corresponding 95% confidence intervals. Statistical analysis was conducted using SPSS IBM version 28, with the alpha significance values set at the 0.05 and 0.01 levels.

## 4. Results

Our sample size was 172 patients. All these patients are residents of the Kingdom of Saudi Arabia, and none of the patients had a prior history of CVD.

Table 1 presents the descriptive analysis of the patients’ sociodemographic characteristics. The majority of the patients were male (62.2%), and 37.8% were female. The mean age of the sample was 59.98 ± 9.26 years, with an age range spanning 40 years. Of the patients, 12.2% were aged 40−49 years, 38.4% were aged 50−59 years, 35.5% were aged 60−69 years, and the remaining 14% were aged 70 years or older. The ethnic backgrounds for these patients were as follows: 84.9% of them were Middle Eastern, 6.4% of them were of Pakistani or Indian descent, and 8.7% of them were from Asian countries, mainly the Philippines. The mean height of the patients was 1.64 ± 0.09 (meters), and the mean body weight (kilograms) was 81.55 ± 15.52 kg. The mean BMI of the patients was 30.35 ± 5.39, with 1.7% considered to be underweight and 14% having a normal BMI. However, most of the patients were considered to be overweight, with 25.6% considered to have class I obesity and 20.3% considered to have class II obesity.

Table 2 displays the descriptive analysis for the patients’ coronary calcium score and laboratory test findings. The mean CAC score for the patients was measured at 138.92 ± 342.54 points. Because of the presence of negative skewness in the CAC score distribution, the median CAC was also found to be 14.5 points, with an inter-quartile range of 106.25 points (25th percentile = 0 and 75th percentile = 106.25). Moreover, it was found that 46.5% of the patients were considered to have a low CAC score (CAC < 10 points), and 53.5% were considered to have a high (CAC ≥ 10 points) CAC score. The patients’ mean fibrinogen level was 3.81 ± 0.82 g/L, and their mean serum liver function test findings were ALT = 21.31± 10.96 unit/L, AST = 19.14 ± 6.62 unit/L, and ALP = 86.69 ± 36.6 units/L. Furthermore, the patients’ mean serum creatinine, calcium, and fasting blood glucose levels were 80.65 ± 36.66 mcmol/L, 2.43 ± 0.54 mmol/L, and 7.67 ± 3 mmol/L, respectively. Their mean HbA1c level was measured at 7.14 ± 1.55%, and their mean serum phosphate level at 1.15 ± 0.16 mmol/L. Moreover, the patients’ mean serum TC, HDL, and LDL levels were 4.36 ± 1.19 mmol/L, 1.28 ± 0.44, and 2.42 ± 0.94 mmol/L, respectively, but their serum TG was 1.48 ± 0.94 mmol/L. The patients’ mean serum TG/HDL ratio was 1.26 ± 1.10 points. Additionally, the patients’ laboratory tests showed mean serum PTH, TSH, HsCRP, and vitamin A levels of 5.31 ± 2.23 pmol/L, 2.57 ± 1.96 pmol/L, 6.99 ± 20.82 mg/L, and 1.79 ± 0.54 mmol/L, respectively; 11% of the patients were considered to have a higher than expected serum vitamin A concentration. In addition, the patients’ mean serum vitamin E level was 37.10 ± 10.93 mmol/L; therefore, 14.5% of them were considered to have higher than expected serum vitamin E levels. The patients’ mean serum vitamin D level was 78.35 ± 28.03 nmol/L. No statistical differences between females and males were found for vitamin A (1.74 ± 0.58 vs. 1.80 ± 0.52; *p*-value = 0.633), vitamin E (41.41 ± 15.99 vs. 37.61 ± 11.78; *p*-value = 0.189), and vitamin D (80.35 ± 31.07 vs. 77.16 ± 26.15; *p*-value = 0.479) levels.

Table 3 presents the bivariate correlations of the patient laboratory findings and sociodemographic factors with their CAC scores. Patient age was positively and significantly correlated with the mean CAC score (r = 0.281, *p* < 0.010), indicating that, on average, older patients tended to have higher CAC scores. Similarly, the mean serum phosphate level showed a significant positive correlation with the mean CAC score (r = 0.281, *p* < 0.010), suggesting that higher serum phosphate levels were associated with higher CAC scores. Likewise, the serum creatinine level exhibited a positive and significant correlation with the mean CAC score, indicating that higher serum creatinine levels predicted higher CAC scores in this patient group. In contrast, the patients’ serum HDL level was significantly negatively correlated with their mean CAC score, i.e., higher serum HDL levels predicted lower CAC scores. The other laboratory findings did not show significant correlations with CAC scores.

Table 4 displays the results of the bivariate analysis comparing the risk of having a high versus low CAC score. A chi-squared test of association revealed that male patients were significantly more likely to have a high CAC score (≥10 points) than female patients (*p* < 0.001). The independent-samples *t*-test showed that patients with a high CAC score were significantly older than those with a low CAC score (*p* < 0.001). Additionally, a chi-squared test of independence found that patients aged 60–69 years or ≥70 years were significantly more likely to have a high CAC score than patients younger than 60 years (*p* < 0.001). The patients’ ethnicity did not affect their risk of having a high CAC score (≥10 points). Furthermore, according to the independent-samples *t*-test, patients with high CAC scores had significantly higher mean HbA1c levels than those with low CAC scores (*p*-value = 0.018). The independent-samples *t*-tests revealed there were no significant differences between patients with low and high CAC scores in terms of their mean TC, HDL, LDL, and TG levels (*p*-value > 0.05 for each comparison). However, patients with low CAC scores (<10 points) had a significantly lower mean TG/HDL ratio than those with high CAC scores (≥10 points) (*p* = 0.035). Patients with low CAC scores also had significantly higher mean blood vitamin A and vitamin E levels than those with high CAC scores, with *p*-values of 0.001 and 0.008, respectively. Finally, there was no significant difference in mean blood vitamin D levels between patients with low and high CAC scores (*p* = 0.243) according to the bivariate independent-samples *t*-test.

**Figure 2:**
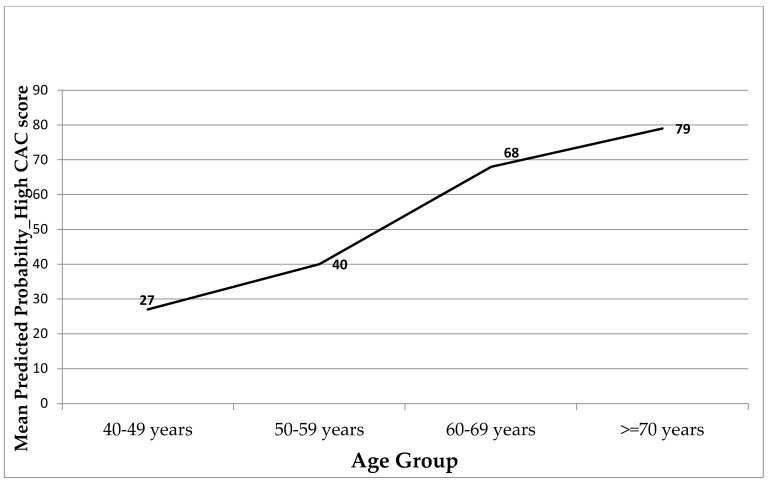
The association between a patient’s age group and their mean predicted probability of having a high coronary artery calcium score (≥10 points).

MBLR was used to ascertain the correlations yielded from the bivariate analysis findings via regression of the patients’ odds of having a high CAC score (CAC ≥ 10 points) against their sociodemographic factors and clinical and laboratory test results. The analysis model results shown in Table 5 reveal that male patients were significantly more likely (3.42 times higher) to have a high CAC score than female patients, with a *p*-value of 0.005. The model also indicates that age was significantly positively correlated with the odds of having a high CAC score. For every additional year of age, the predicted odds of having a high CAC score increased by 1.102 times (or 10.2%), with a *p*-value < 0.001. As shown in Figure 2, as the age of the group increased, the probability of having a high CAC score rose incrementally.

In contrast, ethnicity and BMI did not significantly correlate with the odds of having a high CAC score (CAC ≥ 10 points), with *p*-value = 0.749 and *p*-value = 0.722, respectively. Additionally, the analysis showed that the mean TG/HDL ratio had a positive and significant correlation with the odds of having a high CAC score. With each one-point increase in the TG/HDL ratio, the predicted odds of having a high CAC score increased by 1.654 times (or 65.4%), with a *p*-value = 0.030.

The analysis also indicated that the mean HbA1c level significantly positively impacted the odds of having a high CAC score (OR = 1.364, *p*-value = 0.018). Interestingly, the patients’ mean serum vitamin A level was significantly negatively correlated with the odds of having a high CAC score. For each one-unit increase in serum vitamin A concentration, the odds of having a high CAC score decreased by 85.3% when considering the other variables in the model (*p*-value = 0.002). Lastly, serum vitamin D and E levels, along with other measured independent variables, did not show significant correlations with the odds of having a high CAC score when tested in other iterative competing models.

## 5. Discussion

Our comprehensive study revealed that only vitamin A (among vitamin E and D) was found to be negatively correlated with the CAC score (OR = 0.147), which does not agree with the findings of Hatzigeorgiou et al., who did not find any correlation between vitamin A and the CAC score [16]. Vitamin A’s antioxidant activity may not be sufficient to explain its role in preventing calcification because, in our study, vitamin E was not found to be correlated with the CAC score even though it is also an antioxidant agent. It is well known that arterial calcification is a very complicated process involving multiple factors, including the deposition of lipoproteins, the proliferation of elements in the artery wall, and vascularization with intraplaque hemorrhage [16]. The literature is not clear on the involvement of vitamin A at the cellular level, but vitamin A might be involved in one of these steps; extensive deep cellular level studies are needed. While vitamin A is not well studied in the literature, several studies have investigated whether vitamin E can prevent or treat CAD through its antioxidant properties. Some studies found that vitamin E protected against the oxidation of LDL [17]. In contrast, a systematic review revealed conflicting results between CAC scores and vitamin E levels [18].

Vitamin D’s role in artery calcification is controversial. In a cross-sectional study of 140 individuals without any previous diagnosis of CVD, serum levels of 25(OH)D (a form of vitamin D) were not found to be associated with early-stage CAC [19]. However, another study observed a negative relationship between vitamin D levels and CAC scores, indicating a higher risk of atherosclerosis and the development of calcified and mixed plaques in those with vitamin D deficiency [20]. Interestingly, in a longitudinal study lasting two years, the administration of vitamin D as well as vitamin K did not correlate significantly with CAC scores except for extremely high CAC scores (more than 400) [21]. This might be explained by their magnified impact on the stimulation of the Matrix G protein, which inhibits calcium deposition. In addition, a high CAC score was correlated with low serum vitamin D levels but only in patients with obesity, which has greater impact on cardiovascular health [22]. One of the possible explanations of our results could be the need for both vitamin K and vitamin D in combination in order to have an effect.

Although there is some controversy, it is worth mentioning that our study found no correlation between the levels of TSH or PTH and CAC scores. In contrast, previous studies have reported mixed findings. Some studies suggest that higher TSH levels, particularly in subclinical hypothyroidism (TSH > 7 mIU/L), are linked to an increased risk of CAD [23] and are positively correlated with CAC scores [24,25]. Similarly, while some studies indicate a positive association between serum PTH levels and CAC scores [26], others have found no significant relationship [27]. These discrepancies highlight the need for further research to clarify the role of these hormones in arterial calcification.

Interestingly, a positive correlation between the TG/HDL ratio and the CAC score was found (OR = 1.6). Currently, the TG/HDL ratio is thought to be a very sensitive and specific measure that can predict CVD and metabolic syndromes [28]. However, further studies are needed to predict suitable TG/HDL ratio cutoffs for different ethnicities, genders, diseases, and ages [28]. As a matter of fact, recent studies observed the occurrence of CVD despite a low level of LDL [29,30]. Regarding HDL, even though it is known to play a protective role against CVD due to its antioxidant and anti-inflammatory impact, increasing its level was not correlated with a reduction in the incidence of CVD [31]. Despite the old belief among clinicians that LDL or HDL or TG alone correlate with CVD, it is worth mentioning that due to the complexity of cardiovascular diseases and their integration with other disorders, such as metabolic disorders, a multifactorial holistic approach should be considered when considering the mechanism of the calcification process [32,33]. Regarding TG, new evidence has noted that elevated TG levels are associated with CVD but only when accompanied by a low HDL level [34].

Our analysis model shows that the HbA1c level was positively and significantly associated with the CAC score, (OR = 1.364). This finding is consistent with those of other studies that suggest a similar relationship [35,36] and also supports the sensitivity of using the HbA1c level as a predictor of CVD.

Our comprehensive study revealed that being male is more positively correlated with having a higher CAC score than being female (OR = 3.4). This finding is in line with the studies by Leth et al. and McClelland et al. [37,38]. Interestingly, the mean age of our sample is higher than that of the female premenopausal stage. Therefore, factors other than hormonal status might contribute to the protection mechanism. In addition, age was found to be positively correlated with higher CAC scores, aligning with the general consensus from other studies [15,38,39]. Investigating the factors that have an impact on the CAC score is of great value and requires further investigation, especially as many studies currently support the use of the CAC score as a predictive index for patients at risk of CAD [39].

One of the notable findings in our study is the role of ethnicity. We found no significant correlation between ethnicity and the CAC score, as being Middle Eastern, Pakistani, Indian, or of other Asian descent was not associated with the CAC score. This could be attributed to variations in CAC values across ethnic groups, as reported in previous studies [40,41,42,43], or to the small number of Asian patients in our study. Additionally, the geographic proximity of these populations within Asia may contribute to shared genetic and lifestyle factors. Significant differences might be noticed if other ethnic groups were enrolled, which was not the case in our study. Yet, several studies have noticed significant differences among various ethnic groups. Rosenblatt et al. found that the black ethnic group had higher CAC scores than the Hispanic ethnic group [44]. A study that was conducted in Saudi Arabia in 2015 found higher CAC scores in Saudi women than in US women [42]. This could be due to the obvious ethnic disparity as well as lifestyle variations.

We found that BMI was not correlated with the CAC score, which questions the sensitivity of using BMI as an indicator of CVD. Even though BMI is widely used as an indicator of CVD, recently, other indices have been found to be more sensitive and specific [45,46]. In addition, we did not find a significant correlation between TC, HDL, LDL, and TG and the CAC score, which conflicts with previous studies that found significant correlations [47,48,49,50,51]. This could be due to differences in sample size, ethnic distribution, age, or other factors that reduce the impact of the lipid profile on the CAC score. Additionally, this is an obvious indication that the calcification process is complex and there are many factors affecting it other than the lipid profile.

Due to the high prevalence of premature CAD in Saudi Arabia and its related mortality rate [52,53], as well as the lack of knowledge of its risk factors among the population [54], it is crucial for decision-makers and healthcare providers to focus more attention on the risk factors associated with early atherosclerotic development by employing extensive large, multicenter, and comprehensive studies.

## 6. Perspective for Clinical Practice

Due to the high impact of cardiovascular diseases, not only on patients’ health status but also on the financial and social aspects of life as well as on the entire family, caregivers as well as decision-makers, physicians, nurses, social workers, and patient family members have to take the lead to implement a multidisciplinary prophylactic approach in order to improve patients’ quality of life and minimize disease complications [55]. In addition, the patient has to take more responsibility for controlling and monitoring the progression of the disease [55]. Engagement of the patient and the family members is crucial for better outcomes. An awareness of risk factors such as physical inactivity, an unhealthy diet, stress, and smoking among the younger generation, patients, and caregivers can significantly minimize the incidence as well as the complications of cardiovascular disease [55,56]. Media has to take a pivotal role through educational programs. The newly emerging lifestyle medicine approach has revealed a strong line of defense against attacks from chronic diseases and their risk factors [57]. It is the responsibility of governments and higher level institutions to take the lead in implementing a multidisciplinary approach to achieve the goals.

## 7. Conclusions

The findings of our study indicated the strong impact of vitamin A, the TG/HDL ratio, and HbA1c on the CAC score, among other factors affecting the CAC score, and they need more concern and attention. Understanding the cellular mechanism of vitamin A’s correlation with calcification is of great clinical value. The TG/HDL ratio is emerging as a novel index for CVD when compared to other lipid profile parameters. Therefore, intensive large-scale studies are needed to explore the interpretations as well as cutoff values of this valuable index. Males are more prone to CVD due to their high correlation with the CAC score. Vitamin A administration and strict HbA1c and TG/HDL ratio monitoring could help as prophylactic measures to prevent cardiovascular disease in these patients. These findings could influence specific preventive measures or screening strategies for cardiovascular disease in high-risk populations. A lifestyle medicine approach that includes caregivers as well as patients should be implemented to minimize the incidence and complications of detrimental diseases.

## 8. Limitations

Even though a comprehensive study design was used to assess the correlations of various factors with the CAC score, data regarding some factors could not be obtained through the hospital laboratory tests, such as vitamin K levels, which have been reported to be correlated with the CAC score. In addition to financial support limitations, the relatively small patient sample size and single-center nature of the study are obvious limitations. This study employed a cross-sectional design, which, while providing valuable insights, has some limitations, e.g., preventing the establishment of causality, as data were collected at only a single point in time. In addition, potential confounding variables were not measured in this study, including dietary habits, physical activity levels, and socioeconomic status, which need more objective and extensive assessment tools to be evaluated.

## Figures and Tables

**Table 1:** Descriptive analysis of patients’ sociodemographic characteristics (N = 172).

	Frequency	Percentage
Gender		
Female	65	37.8
Male	107	62.2
Age (years), mean (SD)		59.98 (9.26)
Age group		
40−49 years	21	12.2
50−59 years	66	38.4
60−69 years	61	35.5
≥70 years	24	14
Nationality/Ethnic Group		
Middle Eastern	146	84.9
Indian/Pakistani	11	6.4
Asian	15	8.7
Height (meters), mean (SD)		1.64 (0.09)
Body Weight (kg), mean (SD)		81.55 (15.52)
Body Mass Index (BMI), mean (SD)		30.35 (5.39)
BMI Level		
Underweight	3	1.7
Normal	24	14
Overweight	66	38.4
Obese Class I	44	25.6
Obese class II	35	20.3

SD: Standard deviation, kg: Kilogram.

**Table 2:** Descriptive analysis of the patients’ laboratory test findings and the coronary artery calcium score.

	Mean (SD)
Coronary Artery Calcium (CAC) Score	138.92 (342.54)
CAC Score Level	
0–9 points, n (%)	80 (46.5)
≥10 points, n (%)	92 (53.5)
Serum Fibrinogen Level, g/L	3.81 (0.82)
Serum Alanine Aminotransferase (ALT), Unit/L	21.34 (10.96)
Serum Aspartate Aminotransferase (AST), Unit/L	19.14 (6.62)
Serum Alkaline Phosphatase (ALP), Unit/L	86.69 (36.6)
Serum Creatinine, mcmol/L	80.65 (36.66)
Serum Calcium Level, mmol/L	2.43 (0.54)
Serum Fasting Blood Glucose Level, mmol/L	7.67 (3)
Serum Phosphate Level, mmol/L	1.15 (0.16)
Serum Glycated Hemoglobin (HbA1c) Level (%)	7.14 (1.55)
Serum Total Cholesterol Level (TC), mmol/L	4.36 (1.19)
Serum High-Density Lipoprotein (HDL) Level, mmol/L	1.28 (0.44)
Serum Low-Density Lipoprotein (LDL) Level, mmol/L	2.42 (0.94)
Serum Triglyceride (TG), mmol/L	1.48 (0.94)
TG/HDL Ratio	1.26 (1.10)
Parathyroid Hormone (PTH) Level, pmol/L	5.31 (2.23)
Thyroid-Stimulating Hormone (TSH) Level	2.57 (1.96)
C-Reactive Protein (HsCRP), mg/L	6.99 (20.82)
Vitamin A Level, mmol/L	1.79 (0.54)
Vitamin A Level, mmol/L	
Low to Normal Vitamin A Level	153 (89)
High Vitamin A Level	19 (11)
Vitamin E Level, mmol/L	37.10 (10.93)
Vitamin E Level, mmol/L	
Low to Normal Vitamin E Level	147 (85.5)
High Vitamin E Level	25 (14.5)
Vitamin D Level, nmol/L	78.35 (28.03)

SD: Standard deviation, g/L: gram/liter, mcmol/L: micromole/liter, mmol/L: millimole/liter, pmol/L: picomole/liter.

**Table 3:** Bivariate correlations between the patients’ test findings and the coronary artery calcium score.

Variable	CAC Score
Age (years)	0.281 **
Body Mass Index (BMI)	−0.062
Body Weight (kg)	0.026
Serum Fibrinogen Level, g/L	−0.068
Serum Alanine Aminotransferase (ALT), Unit/L	0.007
Serum Aspartate Aminotransferase (AST), Unit/L	0.087
Serum Alkaline Phosphatase (ALP), Unit/L	0.281 **
Serum Creatinine, mcmol/L	0.212 **
Serum Calcium Level, mmol/L	−0.039
Serum Fasting Blood Glucose Level, mmol/L	0.068
Serum Total Cholesterol (TC) Level, mmol/L	−0.121
Serum High-Density Lipoprotein (HDL) Level, mmol/L	−0.188 *
Serum Low-Density Lipoprotein (LDL) Level, mmol/L	−0.029
Serum Triglyceride (TG), mmol/L	−0.019
TG/HDL Ratio	0.014
Parathyroid Hormone (PTH) Level, pmol/L	−0.030
Thyroid-Stimulating Hormone (TSH) Level	0.073
C-Reactive Protein (HsCRP), mg/L	0.026
Vitamin A Level, mmol/L	−0.085
Vitamin E Level, mmol/L	−0.099
Vitamin D Level, nmol/L	0.007

** Correlation is significant at the 0.01 level (2-tailed). * Correlation is significant at the 0.05 level (2-tailed). g/L: gram/liter, mcmol/L: micromole/liter, mmol/L: millimole/liter, pmol/L: picomole/liter.

**Table 4:** Bivariate analysis of risk factors for having a high versus low coronary artery calcium score.

	CAC Score Level			
Variable	Low (0–9 Points)	High (≥10 Points)	Unadjusted Odds Ratio (95% CI)	*p*-Value
Gender				
Female	43 (53.8)	22 (23.9)	3.698 (1.930:7.084)	<0.001
Male	37 (46.3)	70 (76.1)		
Age (years), mean (SD)	56.34 (7.48)	63.15 (9.52)	1.097 (1.054:1.142)	<0.001
Age group				
40–49 years	14 (17.5)	7 (7.6)	reference group	
50–59 years	38 (47.5)	28 (30.4)	1.474 (0.526:4.129)	0.461
60–69 years	26 (32.5)	35 (38)	2.692 (0.952:7.614)	0.062
≥70 years	2 (2.5)	22 (23.9)	22 (3.986:121.44)	<0.001
Nationality/ethnic group				
Middle Eastern	64 (80)	82 (89.1)	reference group	
Indian/Pakistani	7 (8.8)	4 (4.3)	0.446 (0.125:1.590)	0.213
Asian	9 (11.3)	6 (6.5)	0.520 (0.176:1.538)	0.237
Body Mass Index (BMI) score, mean (SD)	30.62 (5.76)	30.11 (5.10)	0.982 (0.929:1.039)	0.534
Height (meters), mean (SD)	1.63 (0.08)	1.65 (0.10)	17.578 (0.43:747.443)	134
Body Weight (kg), mean (SD)	80.99 (15.26)	82.03 (15.80)	0.98 (0.976:1.019)	0.82
Serum Fibrinogen Level, g/L	3.76 (0.86)	3.84 (0.79)	1.087 (0.667:1.773)	0.682
Serum Alanine Aminotransferase (ALT), Unit/L	21.69 (12.70)	21.04 (9.25)	0.995 (0.944:1.046)	0.695
Serum Aspartate Aminotransferase (AST), Unit/L	19.61 (6.72)	18.74 (6.55)	0.980 (0.897:1.066)	0.394
Serum Alkaline Phosphatase, Unit/L	85.75 (23.85)	87.51 (44.96)	1.001 (0.992:1.012)	0.753
Serum Creatinine, mcmol/L	77.78 (43.1)	83.14 (30.01)	1.003 (0.991:1.012)	0.353
Serum Calcium Level, mmol/L	2.44 (0.73)	2.42 (0.29)	0.910 (0.505:1.639)	0.752
Serum Fasting Blood Glucose Level, mmol/L	7.42 (3.04)	7.88 (2.97)	1.055 (0.951:1.170)	0.315
Serum Phosphates level, mmol/L	1.15 (0.18)	1.15 (0.15)	0.941 (0.152:5.829)	0.948
Serum Glycated Hemoglobin Level (%)	6.84 (1.56)	7.40 (1.51)	1.277 (1.039:1.570)	0.02
Serum Cholesterol Level, mmol/L	4.51 (1.15)	4.23 (1.22)	0.817 (0.629:1.062)	0.131
Serum High-Density Lipoprotein (HDL) Level, mmol/L	1.34 (0.33)	1.24 (0.53)	0.573 (0.266:1.234)	0.155
Serum Low-Density Lipoprotein (LDL) Level, mmol/L	2.57 (0.99)	2.29 (0.88)	0.729 (0.524:1.014)	0.057
Serum Triglyceride, mmol/L	1.385 (0.74)	1.52 (0.75)	1.277 (0.838:1.947)	0.255
Triglyceride/HDL Ratio	1.12 (0.71)	1.39 (0.98)	1.529 (1.015:2.304)	0.035
Parathyroid Hormone level, pmol/L	5.29 (2.34)	5.31 (2.16)	1.003 (0.844:1.192)	0.972
Thyroid-Stimulating Hormone Level	2.39 (1.80)	2.71 (2.09)	1.091 (0.909:1.309)	0.339
C-Reactive Protein, mg/L	5.39 (17.46)	8.12 (22.96)	1.007 (0.987:1.028)	0.498
Vitamin A Level, mmol/L	2.03 (0.64)	1.62 (0.39)	0.211 (0.083:0.540)	0.001
Vitamin E Level, mmol/L	40.65 (11.70)	34.50 (9.70)	0.947 (0.913:0.983)	0.004
Vitamin D Level, mmol/L	75.57 (26.84)	80.67 (28.94)	1.007 (0.995:1.018)	0.243

SD: Standard deviation, g/L: gram/liter, mcmol/L: micromole/liter, mmol/L: millimole/liter, pmol/L: picomole/liter.

**Table 5:** Multivariable logistic binary regression analysis of a patient’s odds of having a high coronary artery calcium score (≥10 points). N = 172.

		95% C.I. for OR		
Variable	Multivariate Adjusted Odds Ratio (OR)	Lower	Upper	*p*-Value
Gender = Male	3.420	1.439	8.127	0.005
Age (years)	1.102	1.053	1.155	<0.001
Nationality	1.124	0.548	2.304	0.749
Body Mass Index (BMI)	1.014	0.938	1.096	0.722
Triglyceride/High-Density Lipoprotein Ratio	1.654	1.051	2.604	0.030
Serum Glycated Hemoglobin Level (HbA1c) (%)	1.364	1.054	1.765	0.018
Vitamin A Level	0.147	0.043	0.498	0.002
Vitamin D Level	2.188	0.760	6.301	0.147
Constant	0.000			<0.001

Dependent outcome variable—having a high CAC score (CAC ≥ 10 points): No/Yes.

## Data Availability

Available from the corresponding author upon reasonable request.

## References

[1] World Health Organization (2019). WHO. Cardiovascular Diseases..

[2] Vedanthan R., Seligman B., Fuster V. (2014). Global perspective on acute coronary syndrome: A burden on the young and poor: A burden on the young and poor. Circ. Res..

[3] Ralapanawa U., Sivakanesan R. (2021). Epidemiology and the magnitude of coronary Artery Disease and acute coronary syndrome: A narrative review. J. Epidemiol. Glob. Health.

[4] Albeladi F., Salem I.W., Zahrani M., Alarbedi L., Abukhudair A., Alnafei H., Alraiqi A., Alyoubi N. (2022). Incidence of coronary artery disease in King Abdulaziz University Hospital, Jeddah, Saudi Arabia, 2019–2020: A retrospective cohort study. Cureus.

[5] Aljefree N., Ahmed F. (2015). Prevalence of Cardiovascular Disease and Associated Risk Factors among Adult Population in the Gulf Region: A Systematic Review. Adv. Public Health.

[6] Arjmand Shabestari A. (2013). Coronary artery calcium score: A review. Iran. Red Crescent Med. J..

[7] Agha A.M., Pacor J., Grandhi G.R., Mszar R., Khan S.U., Parikh R., Agrawal T., Burt J., Blankstein R., Blaha M.J. (2022). The Prognostic Value of CAC Zero Among Individuals Presenting with Chest Pain: A Meta-Analysis. JACC Cardiovasc. Imaging.

[8] Riley R.F., Batchelor W.B., Goldstein J.A., Al-Lamee R., Shah S., Tremmel J.A., Jaffer F., Henry T.D. (2022). The 2021 AHA/ACC Guideline for the Evaluation and Diagnosis of Chest Pain: An Interventionalist’s Viewpoint. J. Soc. Cardiovasc. Angiogr. Interv..

[9] Kaczmarska E., Kępka C., Dzielińska Z., Pracoń R., Kryczka K., Petryka J., Pręgowski J., Kruk M., Demkow M. (2013). What is the optimal cut-off point for low coronary artery calcium score assessed by computed tomography? Multi-Detector Computed Tomography ANIN Registry. Adv. Interv. Cardiol..

[10] Mao S.S., Pal R.S., McKay C.R., Gao Y.G., Gopal A., Ahmadi N., Child J., Carson S., Takasu J., Sarlak B. (2009). Comparison of coronary artery calcium scores between electron beam computed tomography and 64-Multidetector Computed Tomographic Scanner. J. Comput. Assist. Tomogr..

[11] Wongyikul P., Tantraworasin A., Suwannasom P., Srisuwan T., Wannasopha Y., Phinyo P. (2024). Prediction model for recommending coronary artery calcium score screening (CAC-prob) in cardiology outpatient units: A development study. PLoS ONE.

[12] Chin J.C., Maroules C.D., Lin A.H., Graning R.E., Pressley C.R. (2022). Reporting Coronary Artery Calcium on Low-Dose Computed Tomography Impacts Statin Management in a Lung Cancer Screening Population. Fed. Pract..

[13] Han D., Kolli K.K., Gransar H., Lee J.H., Choi S.-Y., Chun E.J., Han H.-W., Park S.H., Sung J., Jung H.O. (2019). Machine learning based risk prediction model for asymptomatic individuals who underwent coronary artery calcium score: Comparison with traditional risk prediction approaches. J. Cardiovasc. Comput. Tomogr..

[14] Ren Y., Li Y., Pan W., Yin D., Du J. (2022). Predictive value of CAC score combined with clinical features for obstructive coronary heart disease on coronary computed tomography angiography: A machine learning method. BMC Cardiovasc. Disord..

[15] Shah A.K., Dhalla N.S. (2021). Effectiveness of some vitamins in the prevention of cardiovascular disease: A narrative review. Front. Physiol..

[16] Hatzigeorgiou C., Taylor A.J., Feuerstein I.M., Bautista L., O’Malley P.G. (2006). Antioxidant vitamin intake and subclinical coronary atherosclerosis. Prev. Cardiol..

[17] Aksu E., Celik E., Dagli M., Tolun F.I., Balcioglu A.S. (2022). Relationship between Oxidative Stress Markers and Presence of Chronic Total Occlusion in Coronary Artery Disease. J. Coll. Physicians Surg. Pak..

[18] Machado A.D., Andrade G.R.G., Levy J., Ferreira S.S., Marchioni D.M. (2019). Association between vitamins and minerals with antioxidant effects and coronary Artery Calcification in adults and older adults: A systematic review. Curr. Pharm. Des..

[19] Rodrigues I.G., Pinho C.P.S., Filho D.S., Leão A.P.D., Oliveira M.C.M., Barbosa G.P., de Siqueira A.A., Bandeira F. (2021). The impact of visceral fat and levels of vitamin D on coronary artery calcification. Rev. Assoc. Med. Bras..

[20] Eyyüpkoca F., Yüksel Y., Altıntaş M.S., Yıldırım O., Koçak A. (2021). Patients with Vitamin D Deficiency Are at Higher Risk of Developing Calcified and Mixed Plaques. E-J. Cardiovasc. Med..

[21] Hasific S., Oevrehus K.A., Lindholt J.S., Mejldal A., Dey D., Dahl J.S., Frandsen N.E., Auscher S., Lambrechtsen J., Hosbond S. (2023). Effects of Vitamin K2 and D Supplementation on Coronary Artery Disease in Men: A RCT. JACC Adv..

[22] Shah N.P., Lu R., Haddad F., Shore S., Schaack T., Mega J., Pagidipati N.J., Palaniappan L., Mahaffey K., Shah S.H. (2024). Relationship between body mass index and cardiometabolic health in a multi-ethnic population: A project baseline health study. Am. J. Prev. Cardiol..

[23] Rosário P.W.S., Calsolari M.R. (2020). Subclinical Hypothyroidism with TSH > 7 mIU/l and ≤10 mIU/l and Coronary Artery Disease. Horm. Metab. Res..

[24] Silva N., Santos O., Morais F., Gottlieb I., Hadlich M., Rothstein T., Tauil M., Veras N., Vaisman M., Teixeira Pde F. (2014). Subclinical hypothyroidism represents an additional risk factor for coronary artery calcification, especially in subjects with intermediate and high cardiovascular risk scores. Eur. J. Endocrinol..

[25] Rhee C.M., Budoff M., Brent G., You A.S., Stenvinkel P., Novoa A., Flores F., Hamal S., Dailing C., Kinninger A. (2022). Serum Thyrotropin Elevation and Coronary Artery Calcification in Hemodialysis Patients. Cardiorenal Med..

[26] Wu G.Y., Xu B.D., Wu T., Wang X.Y., Wang T.X., Zhang X., Wang X., Xia Y., Zong G.J. (2016). Correlation between serum parathyroid hormone levels and coronary artery calcification in patients without renal failure. Biomed. Rep..

[27] Moe S.M., Chen N.X., Newman C.L., Gattone V.H., Allen M.R. (2014). Abstract 17935: Serum parathyroid hormone is associated with valvular calcification but not with aortic root or coronary artery calcification in patients with stage 3 and 4 chronic kidney disease. Circulation.

[28] Kosmas C.E., Polanco S.R., Bousvarou M.D., Papakonstantinou E.J., Genao E.P., Guzman E., Kostara C.E. (2023). The Triglyceride/High-Density Lipoprotein Cholesterol (TG/HDL-C) Ratio as a Risk Marker for Metabolic Syndrome and Cardiovascular Disease. Diagnostics.

[29] Marston N.A., Giugliano R.P., Park J.G., Ruzza A., Sever P.S., Keech A.C., Sabatine M.S. (2021). Cardiovascular Benefit of Lowering Low-Density Lipoprotein Cholesterol Below 40 mg/dL. Circulation.

[30] Dhindsa D.S., Sandesara P.B., Shapiro M.D., Wong N.D. (2020). The Evolving Understanding and Approach to Residual Cardiovascular Risk Management. Front. Cardiovasc. Med..

[31] Averna M., Stroes E., lipid alterations beyond LDL expert working group (2017). How to assess and manage cardiovascular risk associated with lipid alterations beyond LDL. Atheroscler. Suppl..

[32] Rinkūnienė E., Butkutė E., Puronaitė R., Petrulionienė Ž., Dženkevičiūtė V., Kasiulevičius V., Laucevičius A. (2017). Arterial function parameters in patients with metabolic syndrome and severe hypertriglyceridemia. J. Clin. Lipidol..

[33] Nur Zati Iwani A.K., Jalaludin M.Y., Yahya A., Mansor F., Md Zain F., Hong J.Y.H., Wan Mohd Zin R.M., Mokhtar A.H. (2022). TG: HDL-C Ratio as Insulin Resistance Marker for Metabolic Syndrome in Children With Obesity. Front. Endocrinol..

[34] Gu X., Li Y., Chen S., Yang X., Liu F., Li Y., Li J., Cao J., Liu X., Chen J. (2019). Association of Lipids With Ischemic and Hemorrhagic Stroke: A Prospective Cohort Study Among 267 500 Chinese. Stroke.

[35] Scicali R., Giral P., Gallo A., Di Pino A., Rabuazzo A.M., Purrello F., Cluzel P., Redheuil A., Bruckert E., Rosenbaum D. (2016). HbA1c increase is associated with higher coronary and peripheral atherosclerotic burden in non diabetic patients. Atherosclerosis.

[36] Yu J., Gao B. (2021). Nonlinear relationship between HbA1c and coronary artery calcium score progression: A secondary analysis based on a retrospective cohort study. Diabetol. Metab. Syndr..

[37] Leth K.W., Dalgård C., Gerke O., Lindholt J.S., Lambrechtsen J., Frost L., Karon M., Egstrup K., Busk M., Diederichsen A.C.P. (2025). Sex-specific associations between total cholesterol and non-high-density lipoprotein cholesterol and the presence and extent of coronary artery calcifications. Eur. J. Prev. Cardiol..

[38] McClelland R.L., Chung H., Detrano R., Post W., Kronmal R.A. (2006). Distribution of coronary artery calcium by race, gender, and age: Results from the Multi-Ethnic Study of Atherosclerosis (MESA). Circulation.

[39] Tramontano L., Punzo B., Clemente A., Seitun S., Saba L., Bossone E., Maffei E., Cavaliere C., Cademartiri F. (2022). Prognostic Value of Coronary Calcium Score in Asymptomatic Individuals: A Systematic Review. J. Clin. Med..

[40] Cho I., Suh J.-W., Chang H.-J., Kim K.-I., Jeon E.J., Choi S.I., Cho Y.-S., Youn T.-J., Chae I.-H., Kim C.-H. (2013). Prevalence and Prognostic Implication of Non-Calcified Plaque in Asymptomatic Population with Coronary Artery Calcium Score of Zero. Korean Circ. J..

[41] Descalzo M., Vidal-Perez R., Leta R., AlOmar X., Pons-Llado G., Carreras F. (2014). Usefulness of coronary artery calcium for detecting significant coronary artery disease in asymptomatic individuals. Rev. Clin. Esp..

[42] Fathala A., Alreshoodi S., Al Rujaib M., Shoukri M., Al Sergani H., Al Buriki J., Al Sugair A. (2015). Coronary artery calcium score in high-risk asymptomatic women in Saudi Arabia. Ann. Saudi Med..

[43] Ohmoto-Sekine Y., Yanagibori R., Amakawa K., Ishihara M., Tsuji H., Ogawa K., Ishimura R., Ishiwata S., Ohno M., Yamaguchi T. (2016). Prevalence and distribution of coronary calcium in asymptomatic Japanese subjects in lung cancer screening computed tomography. J. Cardiol..

[44] Rosenblatt S., Blaha M.J., Blankstein R., Nasir K., Lin F., Yeboah-Kordieh Y., Berman D.S., Miedema M.D., Whelton S.P., Rumberger J. (2025). Racial and Ethnic Differences in Long-Term Cardiovascular Mortality Among Women and Men From the CAC Consortium. JACC Cardiovasc. Imaging.

[45] Habib S.S., Al-Khlaiwi T., Habib S.M., Al-Khliwi H., AbaAlkhail M.B., Albuhayjan N.A., Aljawini N. (2023). Predictive value of arm circumference (AC) and arm muscle circumference (AMC) with cardiovascular risk in healthy and diabetic males. Eur. Rev. Med. Pharmacol. Sci..

[46] Habib S.S., Al-Khlaiwi T., Butt M.A., Habib S.M., Al-Khliwi H., Al-Regaiey K. (2023). Novel Adiponectin-Resistin Indices and Ratios Predict Increased Cardiovascular Risk in Patients with Type 2 Diabetes Mellitus. J. Saudi Heart Assoc..

[47] Raal F.J., Hovingh G.K., Catapano A.L. (2018). Familial hypercholesterolemia treatments: Guidelines and new therapies. Atherosclerosis.

[48] Miname M.H., Santos R.D. (2019). Reducing cardiovascular risk in patients with familial hypercholesterolemia: Risk prediction and lipid management. Prog. Cardiovasc. Dis..

[49] Chen Y., Chang Z., Zhao Y., Liu Y., Fu J., Zhang Y., Liu Y., Fan Z. (2021). Association between the triglyceride-glucose index and abdominal aortic calcification in adults: A cross-sectional study. Nutr. Metab. Cardiovasc. Dis..

[50] Won K.-B., Park E.J., Han D., Lee J.H., Choi S.-Y., Chun E.J., Park S.H., Han H.-W., Sung J., Jung H.O. (2020). Triglyceride glucose index is an independent predictor for the progression of coronary artery calcification in the absence of heavy coronary artery calcification at baseline. Cardiovasc. Diabetol..

[51] Wang J., Huang X., Fu C., Sheng Q., Liu P. (2022). Association between triglyceride glucose index, coronary artery calcification and multivessel coronary disease in Chinese patients with acute coronary syndrome. Cardiovasc. Diabetol..

[52] Al-Khlaiwi T., Habib S.S., Alshammari H., Albackr H., Alobaid R., Alrumaih L., Sendi F., Almuqbil S., Iqbal M. (2025). Severity and Risk Factors Associated with Premature Coronary Artery Disease in Patients Under the Age of 50 in Saudi Population: A Retrospective Study. J. Clin. Med..

[53] Al-Khlaiwi T., Habib S.S., Bayoumy N., Al-Khliwi H., Meo S.A. (2024). Identifying risk factors and mortality rate of premature coronary artery disease in young Saudi population. Sci. Rep..

[54] Al-Khlaiwi T., Alshammari H., Habib S.S., Alobaid R., Alrumaih L., Almojel A., Sendi F., Almuqbil S., Alkhodair M. (2023). High prevalence of lack of knowledge and unhealthy lifestyle practices regarding premature coronary artery disease and its risk factors among the Saudi population. BMC Public Health.

[55] Caggianelli G., Alivernini F., Chirico A., Iovino P., Lucidi F., Uchmanowicz I., Rasero L., Alvaro R., Vellone E. (2024). The relationship between caregiver contribution to self-care and patient quality of life in heart failure: A longitudinal mediation analysis. PLoS ONE.

[56] Sadiq I.Z. (2023). Lifestyle medicine as a modality for prevention and management of chronic diseases. J. Taibah Univ. Med. Sci..

[57] Cangelosi G., Grappasonni I., Pantanetti P., Scuri S., Garda G., Cuc Thi Thu N., Petrelli F. (2022). Nurse Case Manager Lifestyle Medicine (NCMLM) in the Type Two Diabetes patient concerning post COVID-19 Pandemic management: Integrated-Scoping literature review. Ann. Ig..

